# Aspirin‐Derived Salicyl‐CoA Drives Histone Lysine Salicylation Regulated by CBP and SIRT2

**DOI:** 10.1002/advs.77838

**Published:** 2026-09-16

**Authors:** Facai Zhang, Chuanzan Zhou, Jiaoyan He, Pengpeng Huang, Rong Zhang, Huan Hu, Meng Jiao, Linyi Hu, Feng Liu, Xiaolong Qi, Xiang He, Yunkai Yang, Y Eugene Chinn, Dechao Feng, Dahong Zhang, Qi Zhang

**Affiliations:** ^1^ Urology & Nephrology Center Department of Urology Zhejiang Provincial People's Hospital (Affiliated People's Hospital) Hangzhou Medical College Hangzhou China; ^2^ Life Sciences Institute The MOE Key Laboratory of Biosystems Homeostasis and Protection and Zhejiang Provincial Key Laboratory for Cancer Molecular Cell Biology Zhejiang University Hangzhou Zhejiang China; ^3^ Department of Rheumatology and Immunology Zhejiang Provincial People's Hospital Affiliated People's Hospital Hangzhou Medical College Hangzhou China; ^4^ Department of Pathology The Second Qilu Hospital of Shandong University Jinan China; ^5^ Institutes of Biological and Medical Sciences Suzhou Medical College of Soochow University Suzhou Jiangsu China; ^6^ Institute of Clinical Medicine Research Zhejiang Provincial People's Hospital Hangzhou Medical College Hangzhou Zhejiang China; ^7^ Division of Surgery & Interventional Science University College London London UK

**Keywords:** aspirin, histone, post‐translational modification, salicylation, SIRT2

## Abstract

Aspirin, as the most widely used drug in the world, has been reported to exert antiplatelet, anti‐inflammatory, and anti‐tumor effects. However, apart from the hydrolyzed acetyl group acetylating proteins, and salicylic acid functioning as a classic COX enzyme inhibitor, it is unknown whether the salicylic group of aspirin could participate in the salicylation of histone lysine. Herein, we discover and characterize lysine salicylation (Ksa) in histone and 39 Ksa sites of histone were mapped in 3 cell lines. Ksa could be stimulated by aspirin via direct action and conversion to salicyl‐CoA, and regulated by CBP and SIRT2 in vivo. Multi‐omics analyses reveal associations between histone Ksa enrichment and transcriptional changes in physiologically relevant genes. Moreover, immunohistochemistry of Ksa levels in bladder cancer tissues are associated with aspirin exposure and show a preliminary correlation with recurrence‐related outcomes. Collectively, we discover a new histone mark with potential physiological relevance and may reveal a previously unrecognized mechanism underlying the non‐canonical functions of aspirin.

## Graphical abstract

Aspirin‐derived salicyl‐CoA serves as a previously unrecognized acyl donor for protein lysine salicylation. This study identifies histone lysine salicylation as a reversible epigenetic modification regulated by CBP and SIRT2, expanding the biochemical actions of aspirin beyond its canonical acetylation‐dependent mechanisms and providing a new framework for understanding its diverse biological effects.

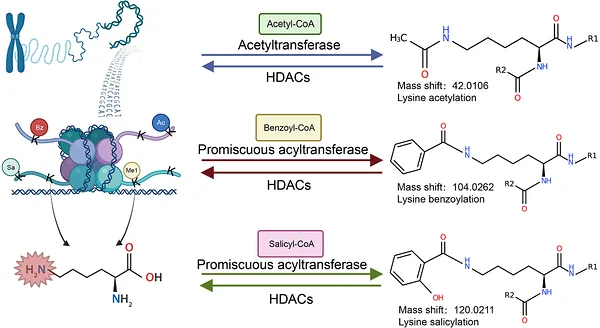

## 1 Introduction

Aspirin is one of the most consumed drugs in the world [1], approximately tens of thousands of metric tons, due to its usage for both analgesia and cardiovascular event prevention [2, 3]. Aspirin is rapidly hydrolyzed by esterase in human body to produce acetate and salicylic acid, which is pharmacologically active for analgesia [4, 5]. Recent studies have shown that it is the acetyl group of aspirin that makes the serine residue (S529) in cyclooxygenase (COX) irreversibly acetylated, which blocks the biosynthesis of prostaglandin G2/H2 (PGG2/H2) and have potent anti‐platelet functions [6, 7]. An Aicardi‐Goutières syndrome study has demonstrated that multiple lysine‐acetylation (K384, K394, K414) of cGAS induced by aspirin could effectively suppress the autoimmunity derived from self‐DNA [8]. Additionally, aspirin‐mediated acetylation at lysine residue (K98) of FGL1 could enhance the immunotherapeutic efficacy in hepatocellular carcinoma [9]. Inspired by these recent findings in epigenomic regulation and the discovery of histone lysine benzoylation [10], we hypothesize that salicylic acid, a 2‐hydroxybenzoic acid, could probably participate in the modification of lysine ε‐NH3 in histone and regulation of chromatin structure and gene transcription.

Posttranslational modification (PTM) in histone could precisely regulate chromosome construction and manipulate the associated genes transcription [11, 12]. The most classic PTM is acetylation in the history of histone acylation, first reported by Allfrey in 1964 [13]. It weakens the interaction between histones and DNA by neutralizing the positive charge on the ε‐amino group of histone lysine, leading to a more open chromatin structure, allowing transcription factors and acetylation readers to be enriched at target genomic loci, and thereby facilitating gene transcription [12]. Methylation could be found in the epsilon‐amino group of lysine or guanidino group of arginine, which could not alter the charge of histone, but the complex of methylation modification is significantly increased [14, 15, 16]. ε‐NH3 in lysine can be mono‐, di‐, and tri‐ methylated, while ω‐nitrogen atoms in arginine could be found mono‐, symmetrically or asymmetrically di‐methylated [17, 18, 19].

Herein, we discovered a new histone mark lysine salicylation (Ksa), which could be found in the individuals administered aspirin or salicylic acid. We comprehensively mapped the modification sites of salicylation in cells. Through a combination of mass spectrometry, immunoblotting/immunofluorescence, flow cytometry, bioinformatic analysis and knockout animal models, we identified the writer and eraser of the total histone salicylation in cells. Furthermore, we applied a multi‐omics approach (CUT&Tag, CUT&RUN‐qPCR, and RNA‐seq) to reveal its preliminary role in modulating gene transcription.

## 2 Methods

### 2.1 Materials and Animals

Pan‐anti‐Ksa antibody was generated by Huabio; anti‐HDAC1‐11 (ET1605‐35, ET1607‐78, ET1610‐5, ET1612‐51, HA723002, ET1701‐66, ET1612‐67, ET1612‐90, ET1706‐36, ET1612‐69, HA500568) antibodies were purchased from Huabio; Pan‐anti‐Kme antibody (ab7315) was purchased from Abcam; Pan‐anti‐acetylation antibody (66289‐1‐Ig) was purchased by Proteintech; Pan‐anti‐Kla antibody (PTM‐1401RM) and Pan‐anti‐Kbz antibody (PTM‐762) was purchased from PTM Bio Inc; anti‐SIRT1‐7 (9475S, 12650S, 5490S, 69786S, 8782S, 12486S, 5360S), anti‐H3 histone (9715L), anti‐H3K27me2 (8173S), anti‐H3K27ac (9728S), anti‐β‐tubulin (2146S), and anti‐CBP (7389S) antibody were purchased from CST. 293T, T24, 5637, A549, and Hela cell lines were purchased from Chinese Cell Bank/Stem Cell Bank (https://www.cellbank.org.cn/). H3K9 non‐modified peptide, H3K9sa peptide, K‐, Kme1‐, Kme2‐, Kme3‐, Kbz‐, Kac‐, Kprop‐, and Kla‐library peptides were synthesized by Shanghai Apeptide Co. and Yuantide Bio‐tech. Standard 2‐hydroxybenzoyl‐CoA was synthesized by ikekule. Aspirin, D4‐aspirin, salicylic acid, and D6‐salicylic acid purchased from MCE. CUT&Tag assay kit (TD904‐01/02) and CUT&RUN Assay kit (HD103‐01) were purchased from Vazyme. Recombinant SIRT2 (31528) and CREBBP (31590) were purchased from ActiveMotif. C57BL wild‐type and SIRT2^−/−^ mice were purchased from Cyagen.

### 2.2 Western Blotting, Dot blotting and Peptide blocking assay

Cells cultured in dishes were washed twice with PBS prior to the addition of RIPA lysis buffer supplemented with protease and HDAC inhibitors. Following a 30‐min incubation on ice with vortexing at 10‐min intervals, the lysates were centrifuged at 12,000 rpm for 30 min at 4°C. The resulting supernatants were collected, heated in a metal bath, and subjected to total protein quantification using a BCA Protein Assay Kit (Beyotime, Shanghai, China). Equal amounts of protein were separated by SDS‐PAGE and transferred onto PVDF membranes. After blocking with 5% lean milk or BSA in TBS/Tween‐20, the membranes were probed with specific antibodies.

The peptide was serially diluted to the desired concentrations, and 2 µL of each dilution was spotted onto a nitrocellulose membrane. After air‐drying, the membrane was blocked with 5% nonfat milk in PBS for 1 h at room temperature, then incubated with primary antibody (diluted in 5% milk) for 2 h at room temperature or overnight at 4°C. The membrane was washed three times with PBS (5 min each), followed by incubation with a fluorescence‐conjugated secondary antibody (diluted in 5% milk) for 1 h at room temperature in the dark. After three additional PBS washes, signals were visualized using the Odyssey Infrared Imaging System.

To validate antibody specificity, a peptide competition assay was performed. Briefly, 1 µg of antibody and 3 µg of peptide were added to 3 mL of 5% lean milk and incubated overnight at 4°C with rotation. Meanwhile, protein samples were subjected to SDS‐PAGE, transferred to membranes, and blocked with 5% nonfat milk. The membrane was then incubated overnight at 4°C with the antibody‐peptide mixture. After washing, the membrane was incubated with secondary antibody for 1 h at room temperature, and signals were visualized.

### 2.3 CRISPR/Cas9‐Mediated Gene Knockout

Gene knockout was accomplished using the CRISPR/Cas9 system. Briefly, single‐guide RNAs (sgRNAs) targeting the gene of interest were designed and inserted into a suitable Cas9 expression vector. The resulting constructs were introduced into cells via standard transfection protocols described by Zhang's lab [20]. The efficiency of genome editing was assessed via RT‐qPCR or western blotting, to confirm the loss of target gene expression. Clonal populations were isolated to generate stable knockout cell lines by puromycin resistance. The expression of CBP, HDAC1‐11, and SIRT1‐7 were validated by qPCR and Western blotting to ensure consistent depletion of the target proteins.

### 2.4 Histones Extraction by Sulfuric Acid

Histones were extracted using an acid extraction method. Cells grown to 80%–90% confluence were washed twice with pre‐warmed PBS, trypsinized, and collected by centrifugation at 800 rpm for 3 min. The cell pellet was washed once with PBS and lysed in 1 mL of whole cell lysis buffer supplemented with protease inhibitor cocktail on ice for 30 min with occasional vortexing. The lysate was centrifuged at 12 000 rpm for 10 min at 4°C. The supernatant (soluble protein) was discarded, and the pellet (insoluble fraction containing histones) was washed once with 1 mL of pre‐chilled PBS by centrifugation at 12 000 rpm for 10 min at 4°C. The pellet was resuspended in 10 volumes of 0.4 N H_2_SO_4_ by gentle pipetting. If clumps persisted, the suspension was briefly sonicated to disrupt the pellet. The sample was incubated overnight at 4°C with rotation to extract histones. After centrifugation at 12 000 rpm for 10 min (20 min if sonicated) at 4°C, the supernatant was transferred to new tubes. Trichloroacetic acid (TCA) was added dropwise to a final concentration of 20%, mixed by inversion, and incubated on ice for at least 30 min (longer if more protein). Precipitated histones were collected by centrifugation at 12 000 rpm for 10 min at 4°C. The pellet was washed three times with 500 µL of ice‐cold acetone (centrifuging at 12 000 rpm for 10 min at 4°C each time). After the final wash, the pellet was air‐dried to remove residual acetone and resuspended in 20–100 µL of distilled water. Histone concentration was determined using a BCA assay.

### 2.5 2‐Hydroxybenzoyl‐CoA Extraction From Cells

This protocol was adapted from a previously published method for intracellular Acetyl‐CoA extraction [21]. Cells were collected and resuspended in 1 mL of ice‐cold 10% TCA. Adherent cells were scraped directly in TCA on ice, while suspension cells were pelleted and then resuspended in TCA. The suspension was transferred to a 1.5 mL tube and sonicated (12 × 0.5 s pulses). After centrifugation at 13 000–17 000 × g for 10 min at 4°C, the supernatant (containing 2‐hydroxybenzoyl‐CoA) was collected. The supernatant was loaded onto a hydrophilic reversed‐phase solid phase extraction column pre‐washed with 1 mL of methanol and equilibrated with 1 mL of water. The column was desalted with 1 mL of water, and 2‐hydroxybenzoyl‐CoA was eluted with 1 mL of methanol containing 25 mM ammonium acetate. The eluate was evaporated to dryness under nitrogen flow. The remaining protein pellet can be used for Western blot analysis.

### 2.6 HPLC‐MS/MS Analysis of 2‐Hydroxybenzoyl‐CoA

The purified sample from above was resuspended in 50 µL of 5% (w/v) 5‐sulfosalicylic acid (in HPLC‐grade water) and transferred to HPLC‐compatible vials or a 96‐well plate for analysis. Separation was performed on a C18 reversed‐phase column connected to an HPLC system operating at a flow rate of 0.2 mL/min. The mobile phase consisted of buffer A (5 mM ammonium acetate in water), buffer B (5 mM ammonium acetate in acetonitrile/water [95:5, v/v]), and buffer C (0.1% formic acid, 80% acetonitrile in water). The gradient program was as follows: 0–2 min, 2% B; 2–5.5 min, 2%–25% B; 5.5–6 min, 25%–100% B; 6–14.5 min, 100% B; followed by a 5‐min wash with 100% C and 5‐min re‐equilibration to 2% B. The HPLC system was coupled to a mass spectrometer operating in positive ion mode. Data were acquired using a data‐independent acquisition (DIA) or targeted method, with the m/z acquisition window covering the masses of precursors and fragments. Samples were maintained at 4°C prior to injection.

### 2.7 HPLC‐MS/MS Analysis of Peptide or Proteomics

For protein and peptide analysis, cellular proteins were separated by SDS–PAGE and visualized by Coomassie Brilliant Blue staining. Selected protein bands or entire gel lanes were excised and subjected to in‐gel digestion using a modified trypsin protocol, as described previously [22]. Briefly, peptides were extracted from the gel pieces with 50% acetonitrile containing 5% formic acid, dried under vacuum, and reconstituted in LC solvent A consisting of 5% acetonitrile, 0.005% heptafluorobutyric acid, and 0.4% acetic acid.

Peptides were separated on a reversed‐phase capillary C18 column (75 µm internal diameter × 12 cm) using an EASY‐nLC 1000 system coupled to an Orbitrap Exploris 480 mass spectrometer (Thermo Fisher Scientific). After column equilibration, peptide samples were loaded onto the analytical column and eluted using a gradient of solvent B consisting of 95% acetonitrile, 0.005% heptafluorobutyric acid, and 0.4% acetic acid. The mass spectrometer was operated in data‐dependent acquisition mode, in which precursor ions detected in full MS scans were selected for MS/MS analysis.

Raw MS data were processed using Proteome Discoverer version 3.1 (Thermo Fisher Scientific) and searched against the UniProtKB Homo sapiens reference proteome database downloaded on September 3, 2023. Trypsin/P was specified as the proteolytic enzyme, allowing up to 4 missed cleavages. Lysine salicylation (+120.0211 Da) was specified as a variable modification.

### 2.8 Immunofluorescence Staining

T24 wild‐type cells, along with CBP‐KO and SIRT2‐KO cells, were seeded onto glass coverslips. Cells attached to the coverslips were rinsed twice with commercial phosphate‐buffered saline, followed by fixation in 4% paraformaldehyde for 15 min at ambient temperature. After two washes with PBS, the specimens were permeabilized with 0.5% Triton X‐100 for 10 min at room temperature and subsequently blocked with 3% BSA for 60 min. The coverslips were then incubated overnight at 4°C with a cocktail of primary antibodies: mouse pan‐anti‐Kac, rabbit pan‐anti‐Ksa (both at 1:100 dilution), and rabbit pan‐anti‐Kme. Following two washes with PBST (PBS containing 0.1% Tween 20), the cells were incubated for 60 min at room temperature with corresponding fluorescent secondary antibodies (all diluted 1:100). Nuclear counterstaining was performed with 4',6‐diamidino‐2‐phenylindole, and the coverslips were mounted onto slides. Fluorescence imaging was conducted using a super‐resolution confocal microscope system.

### 2.9 Immunohistochemical Staining and Immunoreactivity Scoring

Paraffin‐embedded tissue sections were baked at 60°C overnight and subsequently deparaffinized in xylene, followed by rehydration through a graded ethanol series. After washing with PBS, antigen retrieval was performed by heating the sections in antigen retrieval buffer using a microwave oven, followed by cooling and repeated retrieval cycles. Endogenous peroxidase activity was blocked with 3% H_2_O_2_ for 10 min. The sections were then blocked with 1% BSA for 1 h at room temperature and incubated with pan‐anti‐Ksa antibody diluted 1:300 in 1% BSA for 2 h at room temperature or overnight at 4°C. After PBS washing, the sections were incubated with the corresponding secondary antibody for 1 h at room temperature. Immunoreactivity was visualized using DAB, with color development monitored microscopically and terminated with distilled water after approximately 3–5 min. The sections were subsequently counterstained with hematoxylin for 5 min, rinsed with distilled water, mounted with neutral resin, and examined by light microscopy. Representative images were acquired for subsequent evaluation.

The immunoreactivity score (IRS) was calculated by multiplying the staining intensity score (0–3) by the percentage of positive cells score (0–4), yielding a total score ranging from 0 to 12. Staining intensity was scored as follows: 0, no staining; 1, light yellow; 2, yellow‐brown; and 3, dark brown. The percentage of positive cells was scored as follows: 0, 0%–5%; 1, 6%–25%; 2, 26%–50%; 3, 51%–75%; and 4, >75%. The resulting IRS was used to classify samples according to the level of positive staining for subsequent statistical analysis.

### 2.10 Molecular Docking and Molecule Dynamics

HDOCKlite was employed to predict the binding modes between SIRT2 and modified H3 peptides (H3K9ac and H3K9sa). Prior to docking, the structure of SIRT2 was retrieved from the PDB database (ID: 8QT1). The structures of H3K9ac and H3K9sa were predicted using AlphaFold3 (https://github.com/google‐deepmind/alphafold3). All structures were processed with PyMOL 2.5.3 to remove non‐protein components. Global rigid docking was performed using HDOCKlite with default parameters. The top‐ranked binding conformation for each complex was visualized and analyzed using PyMOL 2.5.3.

Based on the protein‐protein complexes obtained from docking as initial structures, all‐atom molecular dynamics (MD) simulations were performed using AMBER 24. Prior to simulation, AM1‐BCC charges for non‐standard amino acids were calculated using the antechamber module. The non‐standard residues and proteins were described by the GAFF2 small molecule force field and ff14SB protein force field, respectively. Hydrogen atoms were added to each system using the LEaP module, and the system was solvated in a truncated octahedral TIP3P water box with a 10 Å buffer distance. Na^+^ or Cl^−^ ions were added to neutralize the system charge. Finally, topology and parameter files were generated for simulation.

Each system underwent energy minimization consisting of 2500 steps of steepest descent followed by 2500 steps of conjugate gradient. After minimization, the system was gradually heated from 0 to 298.15 K over 200 ps under constant volume and temperature coupling. Subsequently, a 500 ps NVT equilibration was performed at 298.15 K to ensure uniform solvent distribution, followed by a 500 ps NPT equilibration. Finally, a 50 ns NPT production run was conducted under periodic boundary conditions. During simulation, the non‐bonded cutoff was set to 10 Å, long‐range electrostatic interactions were handled using the Particle Mesh Ewald (PME) method, hydrogen atom bonds were constrained using the SHAKE algorithm, and temperature was controlled via the Langevin thermostat with a collision frequency of 2 ps^−^^1^. Pressure was maintained at 1 atm, the integration time step was 2 fs, and trajectories were saved every 10 ps for subsequent analysis

### 2.11 Cleavage Under Targets and Tagmentation (CUT&Tag) Assay

The CUT&Tag assay was conducted by Vazyme Biotechnology Co., Ltd. (Nanjing, China) in accordance with established protocols [22]. In brief, approximately 1 × 10^6^ cells were collected, washed extensively, and incubated with Concanavalin A‐coated magnetic beads for immobilization. Bead‐bound cells were then transferred to dig‐wash buffer supplemented with the pan‐Ksa antibody and incubated with gentle shaking. Following washing steps, samples were treated with the pG‐Tn5 transposome and washed again. Tagmentation was performed under controlled temperature in the appropriate buffer, and the reaction was terminated using a stop solution containing Proteinase K, followed by incubation to release fragmented DNA. Purified DNA was amplified by PCR and further purified to construct high‐quality libraries. Sequencing was carried out on an Illumina NovaSeq platform according to the manufacturer's instructions.

### 2.12 CUT&RUN‐qPCR and RT‐qPCR

The CUT&RUN assay was performed using a Hyperactive pA/G‐MNase Kit. Briefly, 0.5–1 × 10^6^ live cells were harvested and bound to Concanavalin A‐coated magnetic beads for 5–10 min at room temperature. After washing, cells were permeabilized with Digitonin‐containing buffer for 5–10 min at RT and incubated with a primary antibody (pan‐Ksa antibody) overnight at 4°C. Following the removal of unbound antibody, cells were incubated with the pA/G‐MNase fusion protein for 1 h at 4°C. After washing, MNase was activated by adding Ca^2^^+^‐containing buffer, and fragmentation was carried out on ice for 1–1.5 h or at 4°C for 1–4 h. The reaction was terminated with Stop Buffer containing EGTA, and DNA fragments were released by Proteinase K treatment at 37°C for 30 min. DNA was then purified using a column‐based method or phenol‐chloroform extraction. Finally, the enriched DNA was analyzed by qPCR using gene‐specific primers.

The mRNA expression levels of STAT6, KIF20A, PDK2, CDC45, and HADH were assessed by quantitative real‐time PCR (qRT‐PCR). Total RNA was extracted from cells using RNA extraction kits (Magen, Shanghai, China) following the manufacturer's instructions. Extracted RNA was subsequently reverse transcribed into cDNA using the RevertAid RT Kit (Thermo Fisher, USA). qPCR analysis was performed with the Maxima SYBR Green qPCR Master Mix (Thermo Fisher) to determine the relative transcriptional levels of the target genes. The primer sequence of certain genes’ promoters was presented below:

1. CDC45: Forward ACGCTAGGCTCTAAAACACCC; Reverse: TCTGGGTAGTGCATTGTGGG;
2. PDK2: Forward CAGTGGGCAAGGACACTTGA; Reverse: TAGTGGGCGATCCTGACCTA;
3. KIF20A: Forward ATCCTGACACCACCTCCACA; Reverse: AACGCGAACAGAAGTCCACA;
4. STAT6: Forward GAGAGGTGAGAGGCTGAACG; Reverse: GGTTCAGGGAGGACTGGGTA;
5. HADH: Forward TTGTTTTGGGTGTTCGCAGTC; Reverse: TGTGCACCGTTGGTTGATGT;

The primer sequence of certain genes’ exons were presented below:

1. CDC45: Forward TTCGTGTCCGATTTCCGCAAA; Reverse: TGGAACCAGCGTATATTGCAC;
2. PDK2: Forward ATGAAAGAGATCAACCTGCTTCC; Reverse: GGCTCTGGACATACCAGCTC;
3. KIF20A: Forward TGCTGTCCGATGACGATGTC; Reverse: AGGTTCTTGCGTACCACAGAC;
4. STAT6: Forward GTTCCGCCACTTGCCAATG; Reverse: TGGATCTCCCCTACTCGGTG;
5. HADH: Forward ACCAGGCAGTTCATGCGTT; Reverse: ACGTGCTTGACGATTATCTTCTT;

### 2.13 Statistical Analysis

Quantitative data are presented as the mean ± SD from at least three independent biological replicates unless otherwise indicated. Statistical analyses were performed using GraphPad Prism 8.3.0. Comparisons between two groups were conducted using Student's t‐test, whereas comparisons among multiple groups were performed using one‐way ANOVA. Survival curves were compared using the log‐rank test. The number of biological replicates, statistical tests, and *P*‐value thresholds are provided in the corresponding figure legends. A *P* value < 0.05 was considered statistically significant.

## 3 Results

### 3.1 Identification, Verification and Mapping of Histone Ksa Induced by Aspirin

**FIGURE 1:**
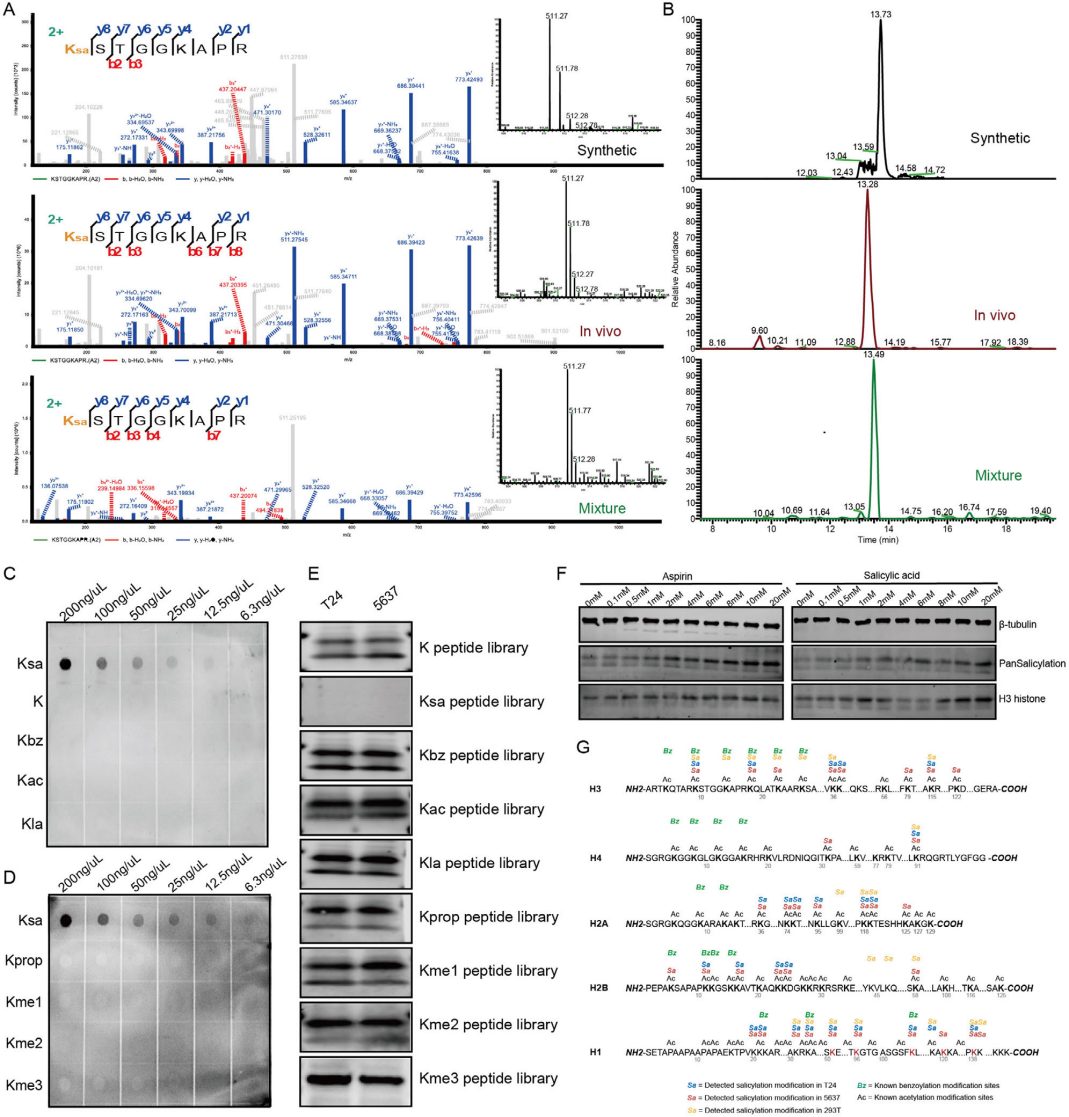
Identification and characterization of histone lysine salicylation. (A) LC–MS/MS spectra of the synthetic H3K9sa peptide, the corresponding cell‐derived peptide, and their mixture, showing consistent precursor m/z values and fragmentation patterns. (B) Extracted‐ion chromatograms of the synthetic H3K9sa peptide, the cell‐derived peptide, and their mixture. The peptides showed comparable retention times and co‐eluted as a single peak in the mixed sample. (C,D) Dot‐blot assays evaluating the specificity of the pan‐Ksa antibody against unmodified lysine and peptides carrying lysine benzoylation, acetylation, lactylation, propionylation, or mono‐, di‐, and trimethylation. (E) Peptide‐blocking assays showing that pre‐incubation with the Ksa peptide library, but not other modified peptide libraries, abolished the pan‐Ksa signals in T24 and 5637 cell lysates. (F) Immunoblotting analysis of histone Ksa levels in cells treated with increasing concentrations of aspirin or salicylic acid. H3 served as the histone loading control, and β‐tubulin was used as the whole‐cell loading control. (G) Distribution of histone Ksa sites identified in T24, 5637, and 293T cells, together with previously reported histone Kbz and Kac sites.

Our initial exploration of protein salicylation modifications derived from 3 acute coronary syndrome patients who suffered from massive hematuria and bladder tamponade after a loading dose of aspirin (300 mg/day). we performed emergency surgery for the tamponade, during which bladder tumors incidentally discovered and resected. Postoperatively, the tumor tissues were subjected to proteomics and PTM analysis using high‐performance liquid chromatography‐mass spectrometry (HPLC‐MS). Intriguingly, we observed that several lysine in peptides were modified with a mass shift of + 120.0211 (Figure S1A–D). For instance, histone H3 peptide, K_+120.0211_STGGK_+120.0211_APR, was identified 2 lysine with a mass shift of +120.0211. Moreover, non‐histone peptide (tryptically digested from SERPINH1), K_+120.0211_PAAAAAPGTAEK_+120.0211_LSPK_+120.0211_AATLAER, was also found the same mass shift in multiple lysine. Similarly, we also discovered the same mass shift of lysine in cells cultured in medium with aspirin or salicylic acid. Given the prevalence of this mass shift and the chemical structure of salicylic acid, we assumed that the addition of salicyl‐group to lysine might be a reasonable modification (Figure 1).

To confirm our hypothesized chemical structure of the lysine mass shift in cells, we chemically synthesized a standard H3K9 peptide with salicylation modification in lysine (H3K9sa), which was identical to H3K9 peptide sequence in vivo. We first used HPLC‐MS/MS to evaluate the synthetical H3K9sa and the counterpart peptide in vivo, and determined whether the two sorts of modified peptide had same chemical structure according to chromatographical elution and fragmental pattern in HPLC‐MS/MS. MS/MS showed that the synthetic H3K9sa in vitro, KsaSTGGKAPR, had similar mass‐to‐charge ratio and fragmentation to its counterpart in vivo (Figure 1A). We mixed the synthetic peptide with the tryptic in vivo peptide and found that they matched perfectly. Additionally, HPLC analysis showed that the synthetic peptide and the in vivo peptide had similar elution times (Figure 1B). We then mixed the two and found that their chromatographic peaks matched very well. Therefore, the HPLC‐MS/MS results preliminarily confirmed the salicylation in lysine as a novel modification.

To further validate histone Ksa modification, we developed a home‐made pan‐anti‐Ksa antibody. Dot‐blot analysis revealed that our pan‐anti‐Ksa antibody could recognize Ksa peptide library successfully, and preclude other non‐salicylation modifications, like methylation, acetylation, benzoylation, lactylation and so forth (Figure 1C,D). Subsequently, peptide blocking assays showed that the pan‐anti‐Ksa antibody binds with high affinity specifically to the Ksa modification, with minimal cross‐reactivity to other modifications (Figure 1E). We applied this modification‐specific antibody to examine the change of modification in cells cultured in aspirin or salicylic acid, and found that Ksa modification could significantly increase along with concentration increment of aspirin or salicylic acid (Figure 1F). Moreover, we evaluated Ksa levels in the liver and kidneys of aspirin‐treated mice by immunohistochemistry (IHC) and western blotting. Ksa was predominantly localized to the nucleus, and its levels increased in a dose‐dependent manner following aspirin treatment (Figure S2A,B).

To identify potential histone Ksa sites, three cell lines—including two bladder cancer cell lines (T24 and 5637) and one non‐tumor‐derived cell line (293T)—were treated with aspirin at concentrations of 1–5 mM for 18–24 h. Subsequently, histones were extracted and subjected to data‐dependent acquisition (DDA)‐based proteomic analysis. In order to increase the coverage of histone Ksa site, histone peptides tryptically digested were enriched with a pan‐anti‐Ksa antibody and subsequently analyzed by mass spectrometry (IP‐MS). Total 39 Ksa sites of histone were detected in 3 cell lines, and the distribution patterns of Ksa sites differed among core histones (Figure 1G). Specifically, Ksa sites were predominantly enriched at the N‐terminal of H3 and H2B histone, whereas they were more dispersed throughout the sequences of H2A and H1 histone. Furthermore, only a few salicylation sites were identified on H4 histone. Compared with the N‐terminal distribution of known lysine benzoylation sites (Kbz), Ksa‐sites pattern might imply different chromatin regulation from Kbz.

To determine whether histone Ksa could be detected under lower aspirin exposure, T24, 5637, and 293T cells were treated with 100 µM aspirin for 24 h and analyzed by HPLC–MS/MS. Bladder tumor tissues from two patients who received a preoperative aspirin loading dose were also examined. A total of 13 histone Ksa sites were identified in these cellular and clinical samples (Figure S3A–L), indicating that histone Ksa remains detectable under lower aspirin exposure and clinically relevant conditions.

### 3.2 Aspirin Could Both Directly Promote Ksa and Stimulate Ksa by Generating Salicy‐CoA

**FIGURE 2:**
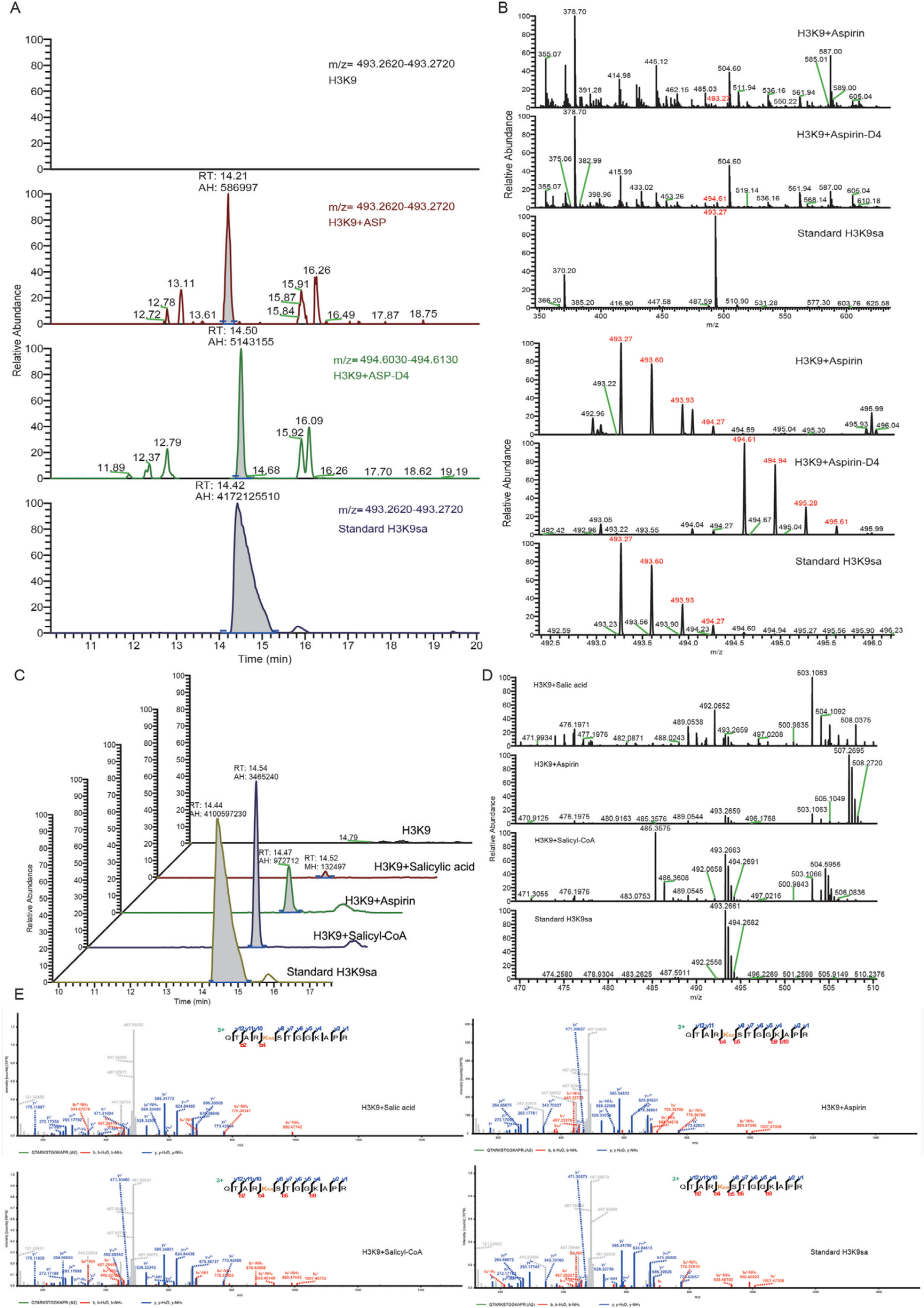
Cell‐free formation of H3K9 salicylation by aspirin, salicylic acid, and salicyl‐CoA. (A) Extracted‐ion chromatograms of the unmodified H3K9 peptide, H3K9 peptide incubated with aspirin or D4‐aspirin, and the synthetic H3K9sa standard. The aspirin‐ and D4‐aspirin‐derived products showed retention times comparable to that of the synthetic H3K9sa peptide. (B) Mass spectra of the products generated by aspirin and D4‐aspirin. The D4‐aspirin‐derived product exhibited the expected approximately +1.34 m/z shift for the triply charged precursor ion, supporting direct transfer of the isotope‐labeled salicyloyl group from aspirin to the H3K9 peptide. (C) Extracted‐ion chromatograms comparing H3K9sa formation after incubation of the H3K9 peptide with salicylic acid, aspirin, or salicyl‐CoA. (D,E) Precursor‐ion and MS/MS spectra confirming that the products generated by the three salicyloyl donors were consistent with the synthetic H3K9sa standard. Under the tested cell‐free conditions, salicyl‐CoA produced the strongest H3K9sa signal, whereas salicylic acid showed the weakest activity.

To elucidate whether aspirin can directly salicylate histone lysine, we chemically synthesized unmodified H3K9 peptides and synthetic H3K9sa standard peptides, and performed in vitro peptide assays. HPLC‐MS‐based analysis revealed that the unmodified H3K9 peptide showed robust Ksa formation after incubation with 1 mM aspirin for 1 h in a cell‐free system (Figure 2A,B). To further validate this reaction, we made in vitro peptide assays again using deuterated aspirin (D4‐aspirin), an isotopic analog of aspirin. Notably, D4‐aspirin also induced salicylation of the unmodified H3K9 peptide, exhibiting a chromatographic retention time identical to that of the ordinary aspirin treatment, with only a +1.34 mass‐charge‐ratio shift detected by MS analysis. Taken together, these results demonstrate that aspirin can directly promote Ksa modification in histone, independent of enzymatic activity or metabolic conversion.

Given that the salicyl moiety transferred to the peptide was derived from aspirin, we next sought to determine whether other salicyl‐containing metabolites could serve as donors for lysine salicylation. To this end, salicylic acid and salicyl‐CoA were added separately to the cell‐free reaction system, and their capacities to induce lysine salicylation were compared with that of aspirin at an equal concentration of 1 mM (Figure 2C–E). Intriguingly, despite containing the same salicyl moiety, salicylic acid exhibited significantly lower efficiency than aspirin in salicylating the lysine residue of the H3K9 peptide. Although the precise reaction mechanism remains unclear, the greater salicylation activity of aspirin than salicylic acid suggests that the ortho‐acetoxy group of aspirin may facilitate transfer of the salicyl moiety to the lysine ε‐amino group. This intrinsic chemical reactivity enables non‐enzymatic acylation of lysine ε‐amino groups easily. Additionally, considering that sodium benzoate could stimulate Kbz by generating benzoyl CoA [10], we compared the salicylation efficacy of salicyl‐CoA with that of aspirin. The salicylation efficacy of salicyl‐CoA exhibited approximately 3.5‐fold higher than that of aspirin in non‐enzyme context, which might imply that salicyl‐CoA is an active form of the salicylic group.

**FIGURE 3:**
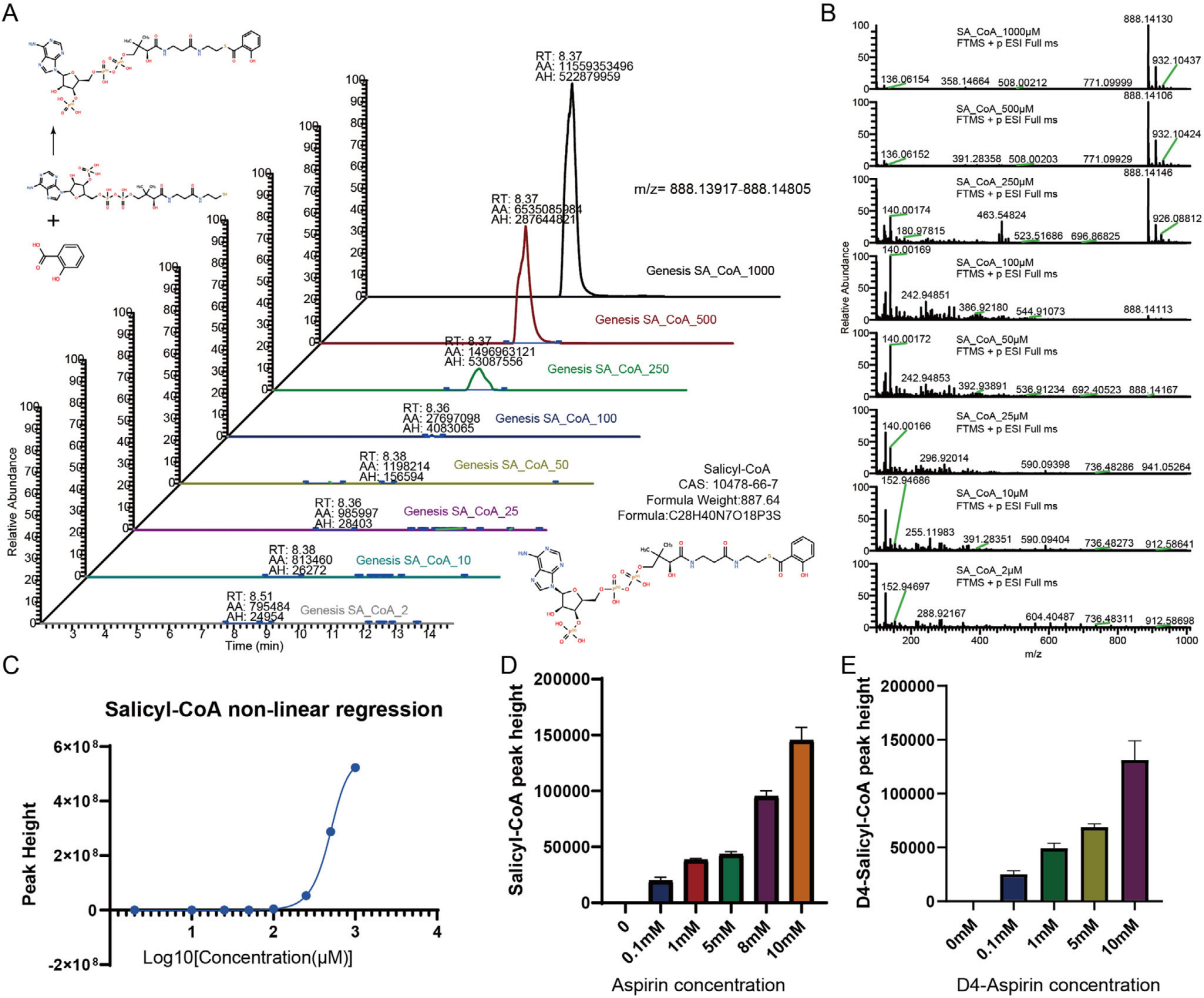
Aspirin is metabolically converted to salicyl‐CoA in cells. (A) Extracted‐ion chromatograms of synthetic salicyl‐CoA standards at the indicated concentrations. The chemical structure and proposed formation of salicyl‐CoA from salicylic acid and CoA are shown. (B) Full‐scan mass spectra of synthetic salicyl‐CoA standards at the indicated concentrations. (C) Nonlinear calibration curve established using the peak heights of synthetic salicyl‐CoA standards. (D) Salicyl‐CoA peak heights measured by LC–MS in cells treated with increasing concentrations of aspirin. (E) D4‐salicyl‐CoA peak heights in cells treated with increasing concentrations of D4‐aspirin, demonstrating the dose‐dependent metabolic conversion of aspirin‐derived salicyloyl groups into salicyl‐CoA. Data in panels D and E are presented as mean ± SD from 3 independent biological replicates.

To investigate whether aspirin could promote Ksa formation through the generation of salicyl‐CoA, we first analyzed a series of salicyl‐CoA standards by small‐molecule LC–MS and correlated their concentrations with the corresponding chromatographic peak heights (Figure 3A,B). These data were then used to generate a nonlinear calibration curve for quantifying salicyl‐CoA concentrations in biological samples (Figure 3C). Then we extracted and measured the cytosolic salicyl‐CoA from cells treated with different concentrations of aspirin (0.1–10 mM aspirin for 24 h). Interestingly, the peak height of salicyl‐CoA raised steadily with the increasing aspirin concentrations (Figure 3D). To further determine whether salicyl‐CoA was directly derived from aspirin, cells were treated with increasing concentrations of D4‐aspirin. LC–MS analysis detected D4‐salicyl‐CoA, and its peak height increased in a dose‐dependent manner with increasing D4‐aspirin concentrations (Figure 3E). These isotope‐tracing results demonstrated that the salicyloyl moiety of intracellular salicyl‐CoA originates directly from aspirin. Furthermore, Salicyl‐CoA was also detected in T24, 5637, 293T, and UM‐UC‐3 cells after treatment with 100 µM aspirin for 24 h, with estimated levels of approximately 28 µM (Figure S4A–C). Taken together, these results demonstrated that aspirin can be metabolized to salicyl‐CoA, which may function as an activated salicyl donor to promote protein lysine salicylation.

### 3.3 CBP Functions as the Acyltransferase to Promote Histone Ksa

**FIGURE 4:**
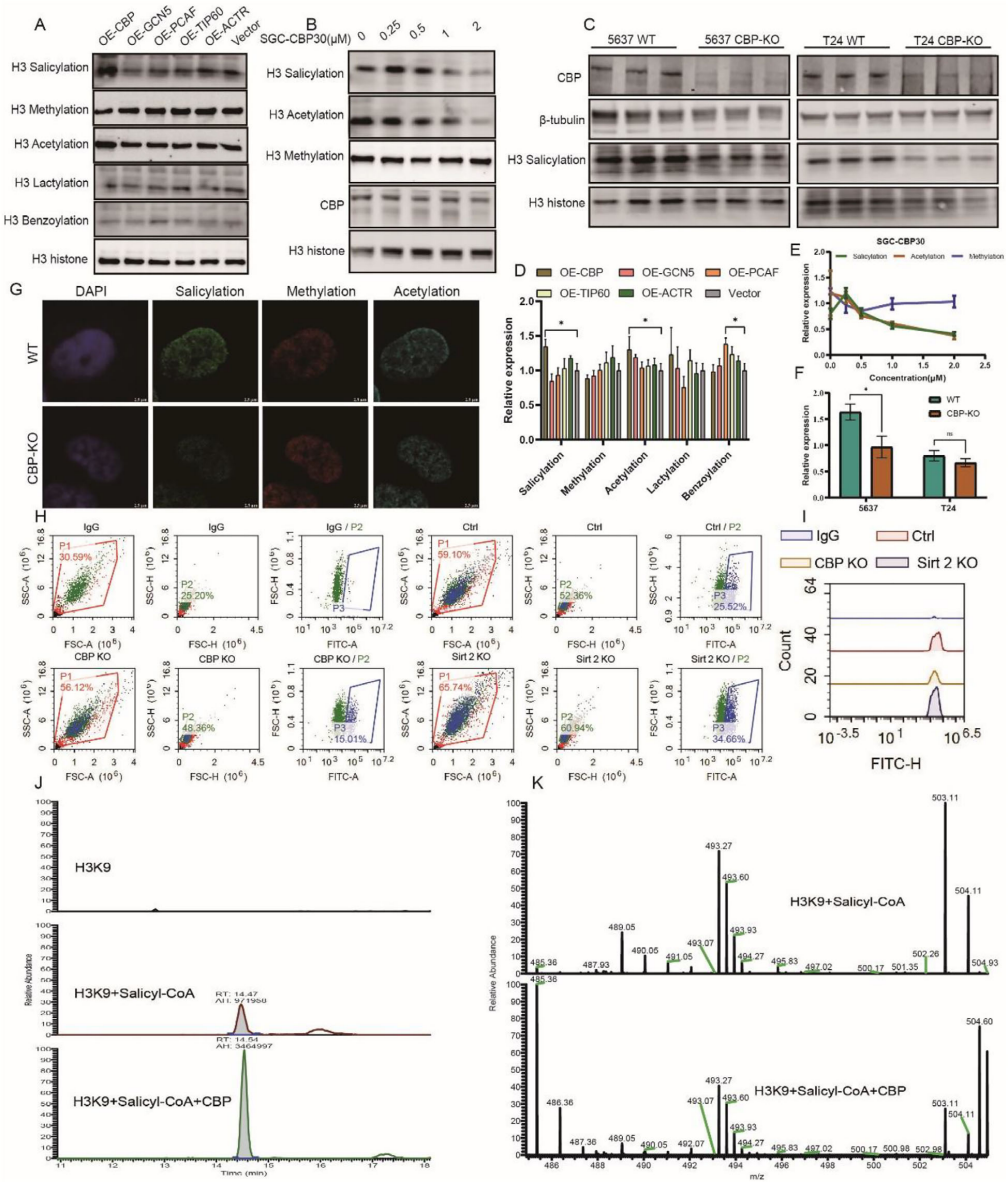
CBP promotes histone lysine salicylation and enhances H3K9sa formation in vitro. (A) Immunoblotting analysis of histone Ksa, methylation, acetylation, lactylation, and benzoylation in cells overexpressing CBP, GCN5, PCAF, TIP60, or ACTR. H3 was used as the loading control. (B) Immunoblotting analysis of histone Ksa and other histone modifications following treatment with increasing concentrations of SGC‐CBP30. (C) Immunoblotting analysis of CBP and histone Ksa in wild‐type and CBP‐knockout 5637 and T24 cells. (D–F) Quantification of the immunoblotting results shown in panels A–C. Band intensities were normalized to H3 and expressed relative to the corresponding control group. (G) Representative immunofluorescence images of Ksa, methylation, and acetylation signals in wild‐type and CBP‐knockout T24 cells. Scale bar, 2.5 µm. (H,I) Representative flow‐cytometry gating strategy and fluorescence distributions of pan‐Ksa staining in control, CBP‐knockout, and SIRT2‐knockout cells. IgG staining was used as the negative control. (J) Extracted‐ion chromatograms of H3K9sa generated from the unmodified H3K9 peptide incubated with salicyl‐CoA in the absence or presence of recombinant CBP. (K) Precursor‐ion spectra of the products generated in the absence or presence of CBP. Recombinant CBP increased H3K9sa formation above the nonenzymatic background produced by salicyl‐CoA alone. Data in panels D–F are presented as mean ± SD from 3 independent biological experiments. Statistical significance was assessed using unpaired two‐tailed Student's t‐test and one‐way ANOVA. ^*^ *p* < 0.05; ns, not significant.

Is aspirin‐induced lysine salicylation an enzymatically regulated post‐translational modification, or is it an irreversible modification once established, similar to N‐terminal acetylation [23]? To identify potential writers of histone Ksa, we examined representative histone acetyltransferases from the p300/CBP, GCN5/PCAF, TIP60, and ACTR/SRC families, several of which have been reported to catalyze noncanonical lysine acylation reactions [24, 25, 26]. Western blot analysis showed that CBP overexpression significantly increased both salicylation and acetylation of histone H3 (Figure 4A,D). Conversely, treatment with the CBP inhibitor SGC‐CBP30 reduced H3 Ksa levels in a concentration‐dependent manner (Figure 4B,E). CBP knockout significantly decreased H3 Ksa levels in 5637 cells and produced a similar, although statistically nonsignificant, downward trend in T24 cells (Figure 4C,F).

Next, we used super‐resolution immunofluorescence microscopy to examine changes in nuclear Ksa signals following CBP knockout in T24 cells. Consistent with the trend observed by western blotting, both histone salicylation and acetylation signals were reduced in the nuclei of CBP‐knockout cells, whereas histone methylation levels remained unchanged (Figure 4G). Flow cytometric analysis further showed a reduced proportion of Ksa‐positive cells after CBP knockout (Figure 4H,I). Taken together, these findings supported a role for CBP in regulating histone lysine salicylation.

To determine whether CBP directly catalyzes lysine salicylation, rather than affecting Ksa indirectly through secondary cellular effects following CBP loss, we performed a cell‐free enzymatic peptide assay followed by LC–MS/MS analysis. CBP markedly increased salicylation of the H3K9 peptide in the presence of salicyl‐CoA, supporting the direct transfer of the salicyl moiety from salicyl‐CoA to the lysine residue (Figure 4J,K). These findings establish CBP as a writer of histone lysine salicylation. To further confirm the direct salicyltransferase activity of CBP, we repeated the cell‐free peptide assay using D4‐salicyl‐CoA. Active CBP markedly increased the formation of D4‐labeled H3K9sa compared with heat‐inactivated CBP or an enzyme‐free reaction containing H3K9 peptide and D4‐salicyl‐CoA (Figure S5A–C). We further compared CBP with GCN5 and TIP60 under the same reaction conditions. CBP exhibited the strongest activity toward H3K9sa formation, whereas GCN5 showed weaker activity and TIP60 showed limited activity (Figure S5D). These results supported CBP as a major salicyltransferase under the tested conditions. Taken together, CBP functions as a major acyltransferase to promote histone Ksa.

### 3.4 SIRT2 Mediates the Removal of Histone Ksa

Histone deacetylases (HDACs), initially characterized as erasers of lysine acetylation, are classified into four major classes [27, 28, 29]. With increasing numbers of brand‐new lysine modification reported, researchers found that SIRT5 could function as the eraser of succinylation (Ksucc), malonylation (Kmal), and glutarylation (Kglu) [22, 30, 31, 32]. SIRT6 demyristoylated ATF2 at K296 and regulates its nucleoplasmic translocation (Kmyr) [33]. Meanwhile, HDAC1 and HDAC 2 was reported to play roles of deacylation of butyrylation (Kbu), crotonylation (Kcr), and lactylation (Kla) [34, 35, 36, 37].

**FIGURE 5:**
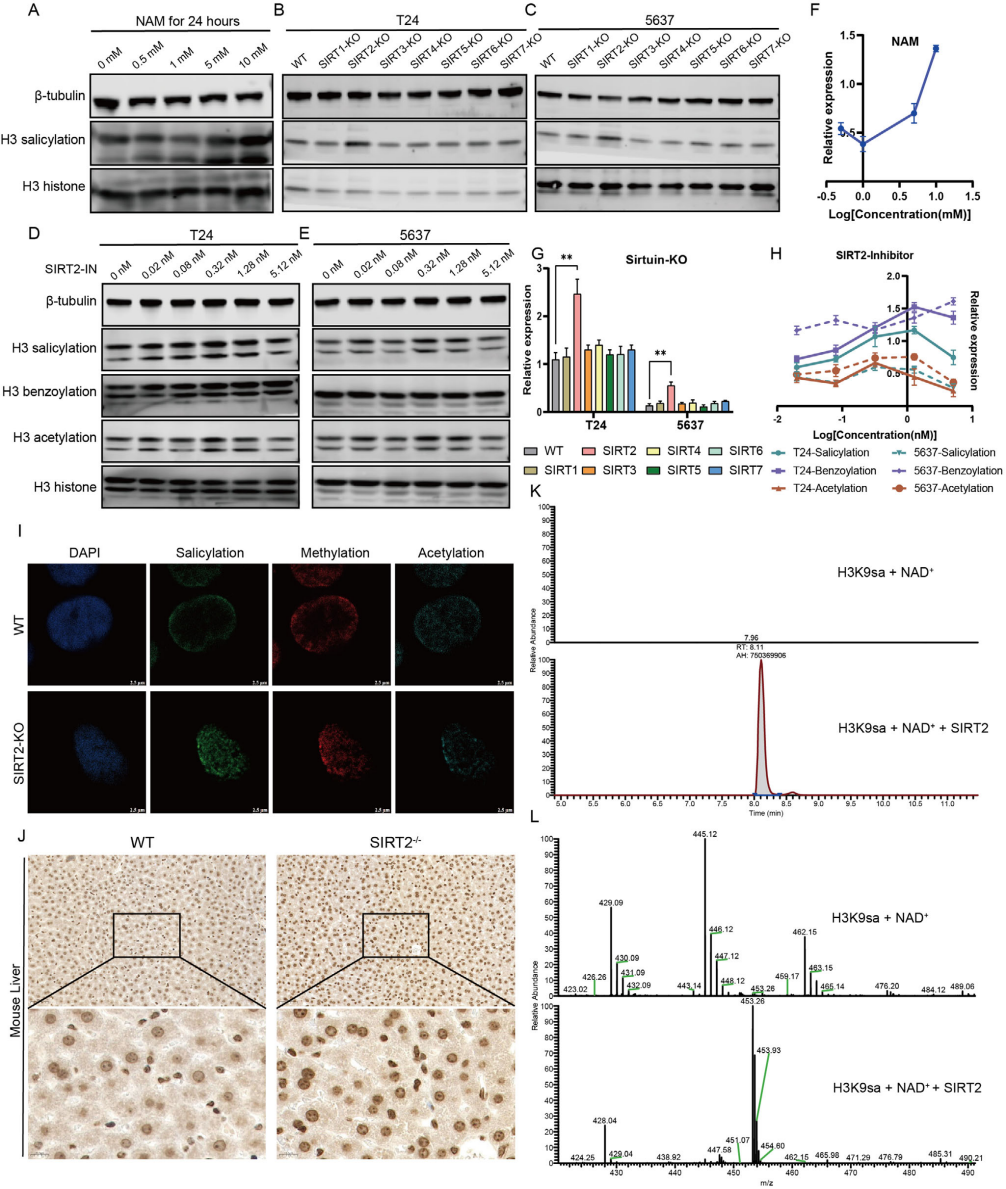
SIRT2 mediates the removal of histone Ksa. NAM, the inhibitor of sirtuin family, could increase Ksa of histone in 293T (A,F). The members of sirtuins’ family were knocked out individually in T24 and 5637, and found that sirt2 was the potent eraser of Ksa (B,C,G). SIRT2_IN, the specific inhibitor for sirt2, promoted the levels of Ksa and Kbz (D,E,H). Immunofluorescence staining showed that sirt2‐ko cells had higher Ksa modification, and the pattern of salicylation was similar to methylation (I). Sirt2‐knockout mice had stronger immunochemical stain administrated with same dosage of aspirin compared with wild type mice (J). HPLC‐MS analysis confirmed that SIRT2 can directly erase salicylation from lysine residues on standard Ksa peptide in vitro (K,L). Data in panels F–H are presented as mean ± SD from 3 independent biological experiments. Statistical significance was assessed using one‐way ANOVA. ^*^ *p* < 0.05; ^**^ *p* < 0.01.

It is the promiscuous deacylase property of the HDACs family that prompted us to explore the potential erasers of histone Ksa. We initially screened for potential Ksa erasers using trichostatin A (TSA), a broad‐spectrum inhibitor of class I, II, and IV HDACs [38]. Western blot analysis showed that H3 Ksa levels decreased only modestly with increasing concentrations of TSA (Figure S6A,B). We then individually knocked out each of the 11 HDAC family members (HDAC1–11) in T24 and 5637 cells. Although deletion of several HDACs increased Ksa levels in one of the two cell lines, no individual HDAC produced a consistent and significant increase in both cell lines (Figure S6C–E). We next treated cells with nicotinamide (NAM), a broad sirtuin inhibitor, to assess whether sirtuins contribute to Ksa removal. Ksa levels increased in a concentration‐dependent manner following NAM treatment, implicating the sirtuin family in the regulation of lysine salicylation (Figure 5A,F). Therefore, we also knocked out SIRT1‐7 individually in T24 and 5637 cells, and surprisingly discovered that SIRT2 could serve as a candidate eraser of Ksa in both T24 and 5637 cells (Figure 5B,C,G), which was also in accordance with the results of sirt2‐specific inhibitors (SIRT2‐IN) (Figure 5D,E,H). Similar effects were observed in A549 and 293T cells, further supporting a role for SIRT2 in regulating Ksa removal across multiple cell types (Figure S6F–H).

Again, super‐resolution microscopy was used to assess nuclear Ksa signals by immunofluorescence in SIRT2‐knockout T24 cells. Consistent with the western blotting results, histone Ksa signals were enhanced in the nuclei of SIRT2‐knockout cells, whereas histone acetylation levels slightly increased (Figure 5I). Notably, in wild‐type cells, the nuclear salicylation signal exhibited a distribution pattern similar to that of methylation marks, being enriched in DAPI‐dense, compact chromatin regions. This spatial correlation hinted at a possible involvement in chromatin‐mediated regulatory processes. Moreover, flow cytometric analysis showed that the proportion of Ksa‐positive cells was significantly higher in SIRT2‐knockout cells than in wild‐type cells (Figure 4H,I). Furthermore, we evaluated the role of SIRT2 in Ksa removal in vivo using SIRT2‐knockout mice (Figure S7). Compared with wild‐type mice receiving the same dose of aspirin, SIRT2^−/−^ mice exhibited significantly higher hepatic Ksa levels, as assessed by immunohistochemistry (Figure 5J).

To determine whether SIRT2 directly catalyzes lysine desalicylation, we performed a cell‐free peptide assay followed by LC–MS/MS analysis. SIRT2 significantly increased the proportion of unmodified H3K9 peptide compared with the enzyme‐free control, indicating direct conversion of H3K9sa to unmodified H3K9 (Figure 5K,L). To further evaluate the specificity of this reaction among sirtuin family members, purified active SIRT1–7 proteins were individually incubated with the H3K9sa peptide under identical conditions. Only SIRT2 efficiently promoted the formation of the corresponding unmodified H3K9 peptide, as confirmed by LC–MS/MS (Figure S8A–C). Taken together, these findings demonstrate that SIRT2 selectively catalyzes the direct desalicylation of H3K9sa in vitro among the tested sirtuin family members.

**FIGURE 6:**
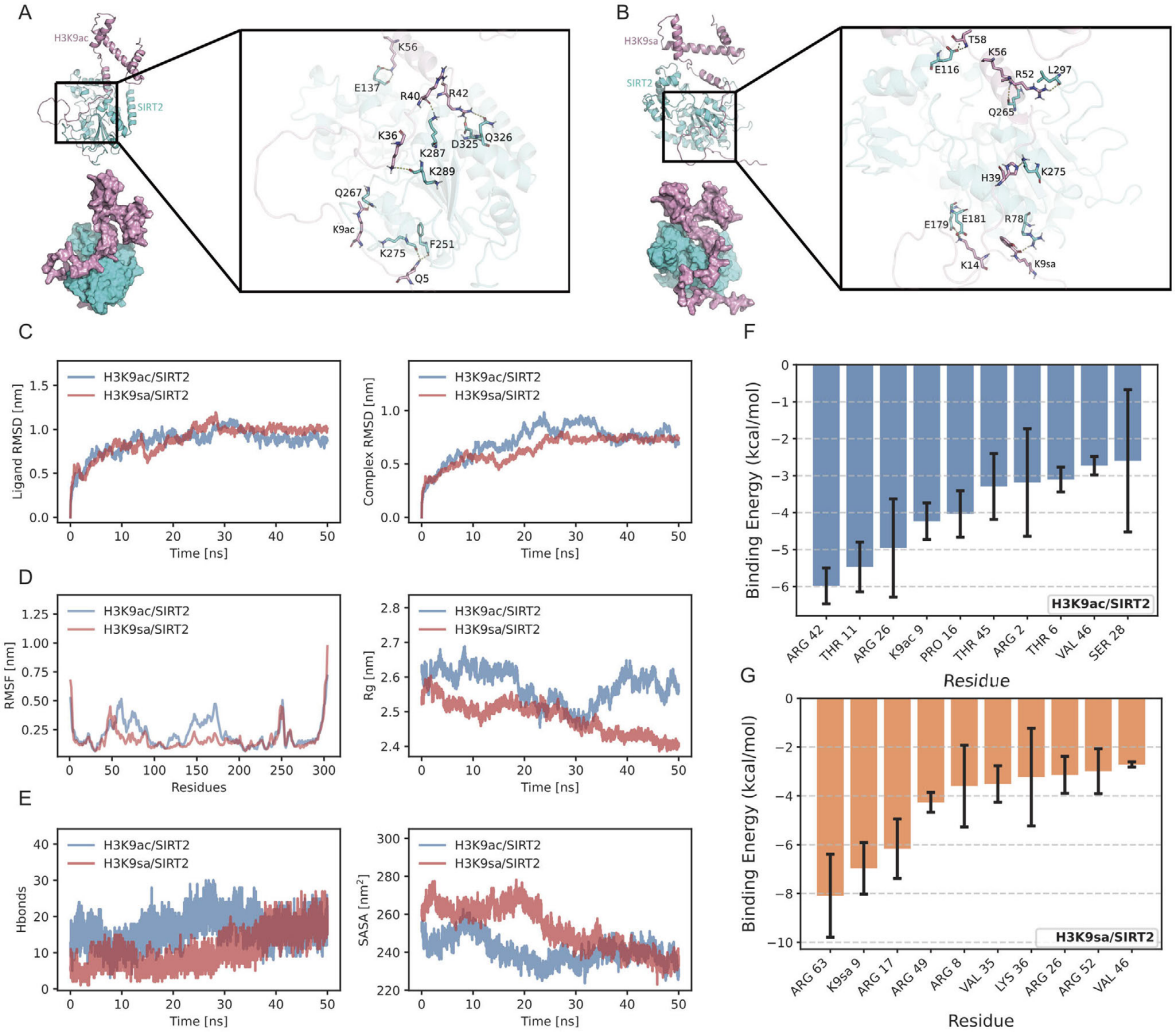
Molecular docking and dynamics simulations suggest favorable recognition of H3K9sa by SIRT2. Similar to the SIRT2‐H3K9ac interaction, SIRT2 formed extensive hydrogen bonds with multiple residues on the H3K9sa, including the modified K9sa (A,B). Compared to the H3K9ac/SIRT2 complex, H3K9sa/SIRT2 complex exhibited lower complex RMSD (C), lower RMSF(D), and a smaller radius of gyration (D). Additionally, it significantly increased hydrogen bonds and markedly reduced SASA (E). The key residues of H3K9sa/SIRT2 complex exhibited stronger overall binding energies (F,G). Collectively, the computational analyses suggested that H3K9sa can be accommodated by the SIRT2 catalytic pocket and may form a relatively compact and stable complex with SIRT2 compared with H3K9ac.

To investigate the interaction between SIRT2 and H3K9sa, we performed molecular docking and molecular simulation, with the SIRT2‐H3K9ac complex serving as a positive control for comparison. Molecular docking revealed that, similar to the SIRT2‐H3K9ac interaction, SIRT2 forms extensive hydrogen bonds with multiple residues on the H3K9sa peptide, including the modified K9sa residue itself (Figure 6A,B). Molecular dynamics (MD) simulations over 50 ns under isothermal‐isobaric ensemble conditions revealed distinct structural dynamics between the two complexes (Figure 6C–G). Compared to the H3K9ac/SIRT2 complex, the H3K9sa/SIRT2 complex exhibited lower complex root‐mean‐square deviation (RMSD) (Figure 6C), lower residue root‐mean‐square fluctuation (RMSF) (Figure 6D), and a smaller radius of gyration (Rg). Additionally, it showed a significantly increased number of hydrogen bonds and a markedly reduced solvent‐accessible surface area (SASA) (Figure 6E) with the process of dynamical simulation. The key residues of the H3K9sa/SIRT2 complex exhibited stronger overall binding energies, supporting its more favorable binding characteristics from an energetic perspective (Figure 6F,G). Collectively, these computational findings demonstrated that H3K9sa formed a more stable complex with SIRT2, offering structural insights into how SIRT2 functions as a desalicylase.

### 3.5 Aspirin‐Induced Histone Ksa is Associated With Transcriptional Changes

**FIGURE 7:**
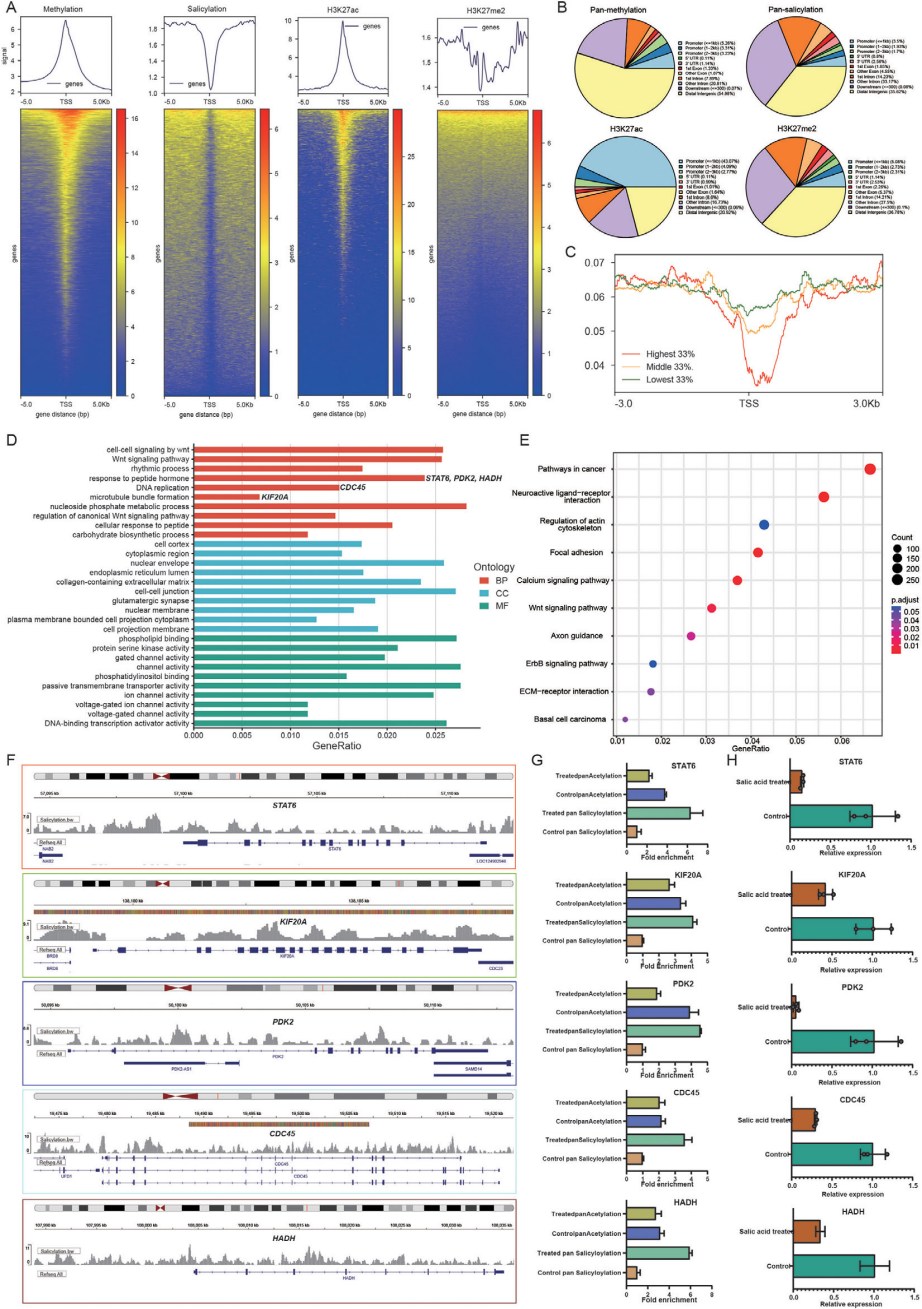
Genomic distribution of histone Ksa and its association with transcriptional changes. (A) TSS heatmaps showing the distributions of pan‐methylation, pan‐Ksa, H3K27ac, and H3K27me2 signals within ±5 kb of transcription start sites (TSSs). In contrast to the prominent TSS enrichment of H3K27ac, pan‐Ksa signals were relatively depleted around TSSs. (B) Genomic distributions of peaks identified using pan‐methylation, pan‐Ksa, H3K27ac, and H3K27me2 antibodies. Pan‐Ksa peaks were predominantly located in distal intergenic and downstream regions, with a smaller proportion located in promoter regions. (C) Pan‐Ksa signals around TSSs of genes stratified into the highest, middle, and lowest expression according to RNA‐seq data. Highly expressed genes showed relatively lower pan‐Ksa signals around TSSs. (D, E) Gene Ontology and KEGG pathway enrichment analyses of genes associated with pan‐Ksa peaks. (F) Representative genome‐browser tracks showing pan‐Ksa enrichment at the STAT6, KIF20A, PDK2, CDC45, and HADH loci. (G) CUT&RUN–qPCR validation of pan‐Ksa enrichment at the indicated gene loci in control and aspirin‐treated cells. Pan‐acetylation was examined in parallel. (H) RT–qPCR analysis of STAT6, KIF20A, PDK2, CDC45, and HADH expression in control and aspirin‐treated cells. Data in panels G and H are presented as mean ± SD from 3 independent biological experiments. Statistical significance was assessed using unpaired two‐tailed Student's t‐test and one‐way ANOVA.

To investigate the epigenetic role of Ksa in transcriptional regulation, we performed CUT&Tag, CUT&RUN‐qPCR, and RNA‐seq analyses in T24 cells. Heatmap analysis centered on transcription start sites (TSSs) revealed that the genomic distribution of Ksa differed from that of the transcriptionally active mark H3K27ac and more closely resembled the pattern of H3K27me2. Specifically, Ksa read density was depleted at the TSS and increased in the surrounding regions within ±5 kb (Figure 7A).

We next performed a genomic distribution analysis of the peaks enriched by pan‐methylation, pan‐salicylation, H3K27ac, and H3K27me2 antibodies. This analysis showed that pan‐salicylation peaks were predominantly located in downstream and distal intergenic regions, with relatively fewer peaks enriched in promoter regions (Figure 7B). The peak distribution pattern of pan‐salicylation was similar to that of H3K27me2 and pan‐methylation, but distinct from that of H3K27ac. To further elucidate the regulatory role of pan‐salicylation on gene expression, we performed an integrative analysis of RNA‐seq data and CUT&Tag enrichment peaks. Interestingly, we found that genes with the highest expression levels (top tertile) corresponded to the lowest read densities at TSS regions, whereas genes with the lowest expression levels (bottom tertile) exhibited relatively higher TSS signals (Figure 7C). These results revealed an inverse association between pan‐Ksa signals around transcription start sites and the expression levels of certain genes.

We next performed Gene Ontology (GO) and Kyoto Encyclopedia of Genes and Genomes (KEGG) enrichment analyses on genes associated with the CUT&Tag peaks. These analyses revealed significant enrichment of biological processes and pathways related to Wnt signaling, peptide hormone responses, DNA replication, and cancer‐associated pathways (Figure 7D,E). Based on promoter‐associated Ksa enrichment, biological relevance, and downregulation in aspirin‐treated cells, we selected STAT6, KIF20A, PDK2, CDC45, and HADH for further validation (Figure 7F). Ksa enrichment at their promoter regions and changes in their transcript levels were subsequently examined by CUT&RUN–qPCR and RT–qPCR, respectively.

CUT&RUN‐qPCR analysis showed significantly increased pan‐Ksa enrichment at the promoter regions of STAT6, KIF20A, PDK2, CDC45, and HADH in aspirin‐treated cells compared with untreated controls (Figure 7G). Consistent with these results, RT–qPCR analysis revealed significantly reduced expression of all five genes following aspirin treatment (Figure 7H). Collectively, these integrated epigenomic and transcriptomic data reveal an inverse association between promoter‐associated Ksa enrichment and the expression of the selected genes.

### 3.6 Ksa Modification in Bladder Cancer Tissue Shows a Preliminary Correlation With Recurrence‐Related Outcomes

**FIGURE 8:**
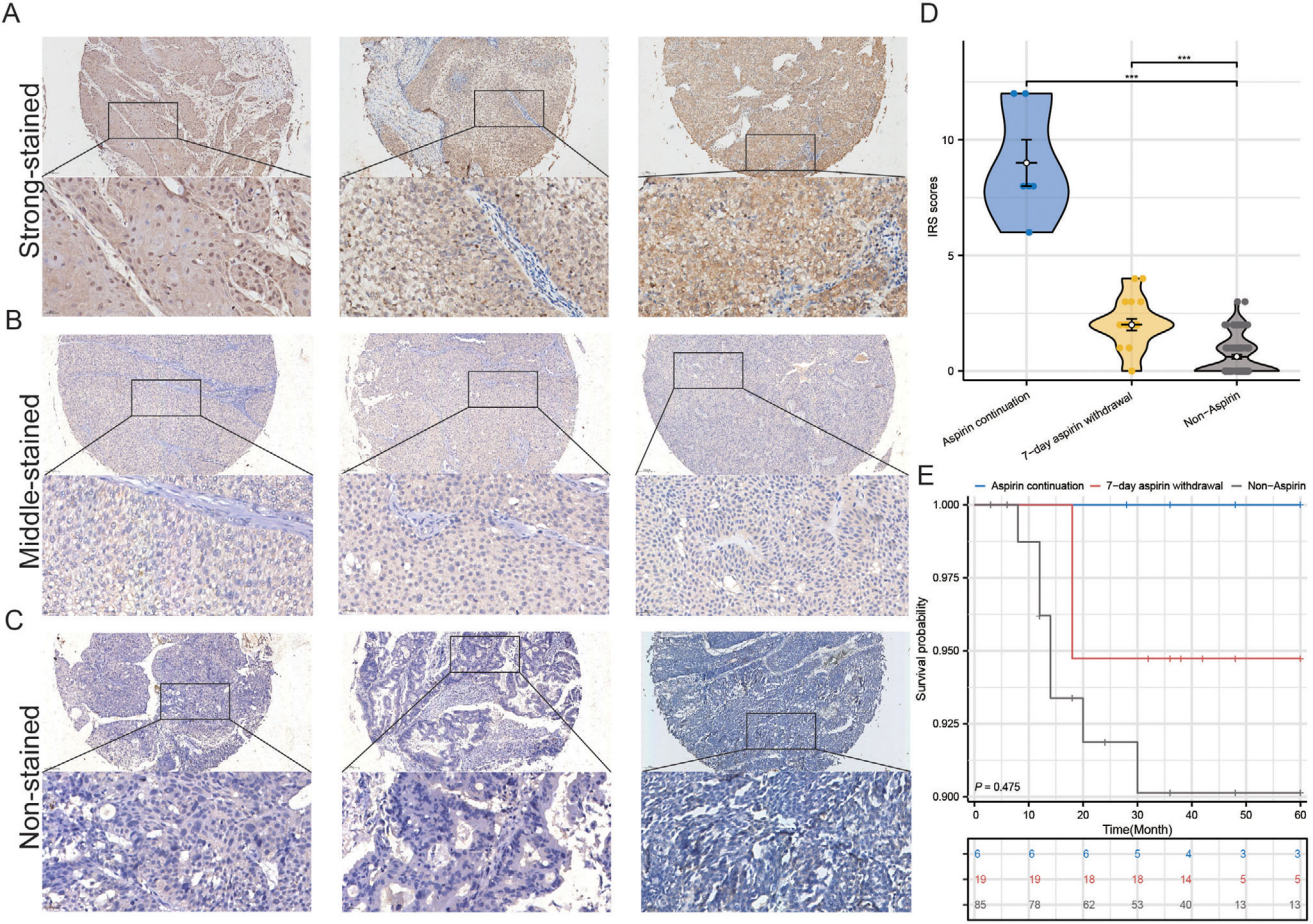
Histone Ksa levels in human bladder tumors according to aspirin exposure and exploratory analysis of recurrence‐free survival. Ksa staining in bladder tumors was significantly elevated in patients receiving a loading dose of aspirin compared to those who had discontinued aspirin or who had never taken it. (A–D) Each point represents one patient. Horizontal lines indicate the median and interquartile range. Statistical significance was assessed using the Kruskal–Wallis test. ^*^ *p* < 0.05, ^**^ *p* < 0.01, ^***^ *p* < 0.001. (E) Kaplan–Meier analysis of recurrence‐free survival in the three patient groups. Differences were evaluated using the log‐rank test. No statistically significant difference was observed among the groups (*p* = 0.475).

Given that pan‐salicylation can be detected by IHC staining and that our HPLC‐MS analysis revealed the presence of Ksa in bladder tumors from patients orally administered aspirin, we sought to investigate the correlation between Ksa levels and recurrence‐related outcomes after transurethral resection of bladder tumor (TURBT). We first performed IHC staining on bladder cancer tissues collected from three groups of patients at our center: (1) patients with incidental bladder cancer who received a loading dose of aspirin for myocardial infarction (300mg as a single dose, marked as aspirin continuation in Figure 8); (2) bladder cancer patients who discontinued aspirin 7 days prior to surgery (100mg as a maintenance dose, marked as aspirin 7‐day aspirin withdrawal in Figure 8), and (3) bladder cancer patients who did not take aspirin (marked as non‐aspirin in Figure 8). The staining results were evaluated using the immunoreactivity score (IRS).

Interestingly, we found that Ksa staining in bladder tumors was significantly elevated in the aspirin loading‐dose group. In the patients who discontinued aspirin, the Ksa levels were lower than those in the loading‐dose group, but remained significantly higher than that in patients who did not take aspirin (Figure 8A–D). Additionally, we performed follow‐up on the 110 patients who underwent TURBT. Kaplan–Meier analysis suggested a nonsignificant trend toward improved recurrence‐free survival in aspirin‐exposed patients; however, no statistically significant difference was observed among the three groups (log‐rank P = 0.475). Given the limited cohort size, postoperative adjuvant therapy and the exploratory nature of this analysis, these findings require validation in larger independent cohorts [39, 40, 41, 42] (Figure 8E). Taken together, our IHC results and follow‐up survey demonstrated that Ksa modification in bladder cancer had a preliminary correlation with recurrence‐related outcomes.

## 4 Discussion

With the rapid advancement of mass spectrometry technology, an increasing number of histone modifications associated with short‐chain endogenous small‐molecule metabolites have been discovered. Modifications such as lactylation [43, 44, 45], propionylation [46, 47], butyrylation [48, 49], crotonylation [50], and succinylation [51] have all been demonstrated to participate in transcriptional regulation and metabolic responses by modulating chromatin structure. In contrast to traditional small‐molecule metabolites, in 2019, Huang discovered benzoylation modification, which contains an aromatic acyl group, and confirmed that it can be dynamically regulated by endogenous enzymes, thereby influencing chromatin structure and gene expression [10]. Inspired by the discovery of benzoylation, we serendipitously identified 2‐hydroxybenzoylation in tumor tissues from bladder cancer patients orally administered with aspirin. By leveraging the novel modification identification strategy previously established by Zhao [10, 52], we have preliminarily and systematically elucidated that the salicylic group of aspirin can also serve as a modifying group for protein lysine residues, besides directly inhibiting COX‐1/2 activity and inducing protein acetylation [6, 7].

The distribution of the 39 identified histone Ksa sites was not uniform. Ksa sites were preferentially enriched in the N‐terminal regions of H3 and H2B, whereas they were more broadly distributed across H2A and H1, and only a few sites were detected on H4. These findings suggest that histone Ksa may exhibit regional preferences rather than random deposition. Moreover, the distribution of Ksa was partially distinct from that reported for histone benzoylation, indicating that the salicyloyl and benzoyl groups may have different structural or enzymatic preferences. Furthermore, proteomic analysis also detected Ksa on non‐histone proteins, including SERPINH1, suggesting that Ksa may represent a broader protein modification. Non‐histone Ksa may potentially affect protein stability, localization, interactions, or activity, but its biological functions require proteome‐wide mapping and site‐specific validation.

Our preliminary study suggests that aspirin can directly mediate salicylation of protein lysine residues in vitro. Interestingly, although salicylic acid lacks the acetyl group present in aspirin, resulting in a significantly reduced efficiency for lysine salicylation in vitro, treatment of cells with salicylic acid still leads to a marked increase in protein salicylation in vivo (Figure 1F). This finding suggests that, within the cellular context, the salicylic group may be converted into salicyl‐CoA—a form with higher acylation efficiency—to participate in enzymatic reactions. Isotope tracing with D4‐aspirin further confirmed that intracellular salicyl‐CoA was directly derived from aspirin. However, the specific enzyme responsible for salicyl‐CoA synthesis was not identified in the present study and remains to be determined through further metabolic and genetic screening. Furthermore, we observed that intracellular salicyl‐CoA levels increased in a dose‐dependent manner with rising aspirin concentrations in the culture medium, confirming that the aforementioned metabolic conversion indeed occurs within cells. However, the nonlinear calibration curve showed relatively limited changes in peak height at salicyl‐CoA concentrations below approximately 25 µM. Therefore, values estimated near this lower range should be interpreted as approximate or semi‐quantitative rather than precise absolute concentrations.

Both direct non‐enzymatic salicylation by aspirin and salicyl‐CoA‐dependent enzymatic transfer can generate Ksa, but their relative contributions in intact cells cannot be quantitatively resolved from the present data. The greater activity of salicyl‐CoA in the cell‐free assay reflects its higher intrinsic efficiency as a salicyloyl donor under the tested conditions but does not establish that the metabolic route predominates in vivo. Additionally, we identified CBP as an acyltransferase capable of utilizing salicyl‐CoA to efficiently catalyze lysine salicylation. Further isotope‐labeled donor experiments and comparisons with other acyltransferases demonstrated that CBP exhibited the strongest salicyltransferase activity among the tested enzymes. Nevertheless, the detectable in‐vitro activity of GCN5 suggests that CBP may not be the only enzyme capable of utilizing salicyl‐CoA.

Several initial discovery and mechanistic experiments in this study employed millimolar concentrations of aspirin or salicylic acid to facilitate the detection and characterization of a previously unrecognized and relatively low‐abundance modification. These experimental conditions should not be interpreted as direct simulations of routine low‐dose aspirin pharmacokinetics. Following oral administration of low‐dose aspirin at 75–100 mg, peak plasma concentrations of intact aspirin are generally approximately 5–6 µM, whereas salicylic acid reaches approximately 20–30 µM [53]. In contrast, high‐dose aspirin historically used for anti‐inflammatory treatment at 3–5 g per day can produce plasma salicylate concentrations in the approximately 1–2 mM range [54]. Importantly, we detected 13 histone Ksa sites in cells exposed to 100 µM aspirin and in bladder tumor tissues obtained from patients receiving a clinically administered aspirin loading dose. Salicyl‐CoA was also detectable in four cell lines following treatment with 100 µM aspirin. These observations support the occurrence of aspirin‐associated histone Ksa under substantially lower exposure conditions. Nevertheless, plasma concentrations do not necessarily reflect intracellular aspirin, salicylic acid, or salicyl‐CoA concentrations, and further quantitative pharmacokinetic studies will be required to define the abundance and persistence of Ksa during long‐term low‐dose aspirin treatment [55].

Similar to the eraser of benzoylation [10], we also identified SIRT2 as the primary enzyme responsible for removing lysine salicylation, at least at the histone level. However, other site‐specific Ksa erasers may also exist and could be masked in analyses of global salicylation levels. Additional Ksa regulators may be identified through broader gain‐ and loss‐of‐function screening of acyltransferase and deacylase families, followed by biochemical validation using purified enzymes and site‐specific Ksa substrates. Although our biochemical and computational results support the direct recognition and removal of H3K9sa by SIRT2, the precise catalytic residues and structural regions responsible for this activity were not determined by mutagenesis or truncation analysis. In addition, quantitative comparisons with other acylated substrates will be required to fully define the substrate preference of SIRT2.

Through integrated analysis of the CUT&Tag and RNA‐seq, we found that genes whose expression was associated with CBP/SIRT2‐mediated histone salicylation were enriched in the items related to Wnt signaling, response to peptide hormone, DNA replication, and pathways in cancer. To validate Ksa's potential regulation, we selected some genes enriched by the Pan‐anti‐salicylation antibody and confirmed Ksa's transcriptional regulation by using CUT&RUN‐qPCR and RT‐qPCR. The similarity between the genomic distributions of pan‐Ksa and H3K27me2 suggests that Ksa may be associated with repressive chromatin contexts. However, the present data do not demonstrate direct co‐localization or functional crosstalk with H3K27me2.

Although the global pan‐Ksa profile showed an inverse association with the expression of selected genes, this finding should not be interpreted as evidence that all histone Ksa sites exert uniform transcriptional effects. Because the pan‐Ksa antibody recognizes multiple salicylated lysine residues, distinct Ksa sites may have divergent or context‐dependent functions. The present analyses cannot resolve the site‐specific functions of individual Ksa marks. Future studies using site‐specific detection and manipulation approaches will be required to establish their direct effects on gene expression and cellular phenotypes.

Ksa was chemically distinct from established histone acylations and displayed a genomic distribution different from that of H3K27ac, but the present findings did not establish that Ksa functions as a completely independent chromatin mark. The partial similarity between pan‐Ksa and methylation‐associated genomic patterns suggested that Ksa may be integrated into a broader histone modification landscape. Ksa might cooperate with, compete with, or be influenced by acetylation and methylation at shared or neighboring lysine residues. Combinatorial chromatin profiling will be required to determine how Ksa interacts with other histone modifications.

Finally, we returned to the clinics that inspired this discovery—bladder cancer tissues—and observed that patients with higher levels of salicylation exhibited a nonsignificant trend toward better RFS following standard TURBT than those without detectable salicylation, suggesting a potential association between histone salicylation and recurrence outcomes [56, 57]. Future studies should further evaluate the clinical relevance of histone Ksa in larger independent bladder cancer cohorts and in additional tumor types. In particular, cancers in which aspirin has shown preventive or therapeutic benefits, such as colorectal cancer, breast cancer, and prostate cancer [58, 59, 60, 61], may provide informative settings for determining whether Ksa is associated with aspirin responsiveness, tumor progression, or clinical outcomes. Collectively, this study provides an innovative epigenetic perspective by preliminarily demonstrating that aspirin intake leads to protein salicylation in vivo. This finding offers a new conceptual framework for future investigations into the multiple pharmacological effects of aspirin, including its anti‐inflammatory [62, 63], anti‐tumor [56, 57], and anti‐aging [64, 65] properties.

## Author Contributions

Conceptualization: F.C. Zhang, Q. Zhang, D.H. Zhang; Methodology: F.C. Zhang, C.Z. Zhou, R. Zhang, D.C. Feng; Software: F.C. Zhang, R. Zhang, Huan Hu; Formal analysis: F.C. Zhang, C.Z. Zhou, J.Y. He, P.P. Huang; Data curation: F.C. Zhang, P.P. Huang, R. Zhang, H. Hu; Validation: Y.K. Yang, R. Zhang, X.L. Qi, F. Liu, M. Jiao, X. He,; Investigation: C.Z. Zhou, J.Y. He, M. Jiao; Resources: C.Z. Zhou, J.Y. He, Q. Zhang, D.H. Zhang; Visualization: F.C. Zhang; Writing – original draft: F.C. Zhang, C.Z. Zhou, R. Zhang, H. Hu, D.C. Feng; Supervision: D.H. Zhang, Q. Zhang, D.C. Feng, Eugene Chinn; Funding acquisition: F.C. Zhang, Q. Zhang, L.Y. Hu, F. Liu; Project administration: Q. Zhang, F.C. Zhang, Eugene Chinn.

## Ethics Approval

This study was approved by the Institutional Review Board of Zhejiang Provincial Peoples’ Hospital (Approval No.: 20260120008). All procedures involving human participants were performed in accordance with the Declaration of Helsinki. Written informed consent was obtained from all patients prior to sample collection. All animal experiments were approved by the Institutional Animal Care and Use Committee of Zhejiang Provincial Peoples’ Hospital (Approval No.: A20260214001) and complied with the ARRIVE guidelines.

## Conflicts of Interest

The authors declare that no conflicts of interest.

## Supporting information




**Supporting File 1**: advs77838‐sup‐0001‐SuppMat.jpg.

**Supporting File 2**: advs77838‐sup‐0002‐SuppMat.jpg.

**Supporting File 3**: advs77838‐sup‐0003‐SuppMat.jpg.

**Supporting File 4**: advs77838‐sup‐0004‐SuppMat.jpg.

**Supporting File 5**: advs77838‐sup‐0005‐SuppMat.jpg.

**Supporting File 6**: advs77838‐sup‐0006‐SuppMat.jpg.

**Supporting File 7**: advs77838‐sup‐0007‐SuppMat.jpg.

**Supporting File 8**: advs77838‐sup‐0008‐SuppMat.jpg.

## Data Availability

The data that support the findings of this study are available on request from the corresponding author. The data are not publicly available due to privacy or ethical restrictions.
